# Daytime Radiative Cooling: A Perspective toward Urban Heat Island Mitigation

**DOI:** 10.1021/acsenergylett.3c00905

**Published:** 2023-07-05

**Authors:** Ioannis Kousis, Roberto D’Amato, Anna Laura Pisello, Loredana Latterini

**Affiliations:** † Environmental Applied Physics Lab (EAPLAB) at Interuniversity Research Center on Pollution and Environment (CIRIAF), University of Perugia, Via G. Duranti 63, Perugia 06125, Italy; # Nano4Light-Lab, Department of Chemistry, Biology and Biotechnology, University of Perugia, Via Elce di Sotto 8, Perugia 06123, Italy; ‡ Department of Engineering, University of Perugia, Via G. Duranti 97, Perugia 06125, Italy

## Abstract

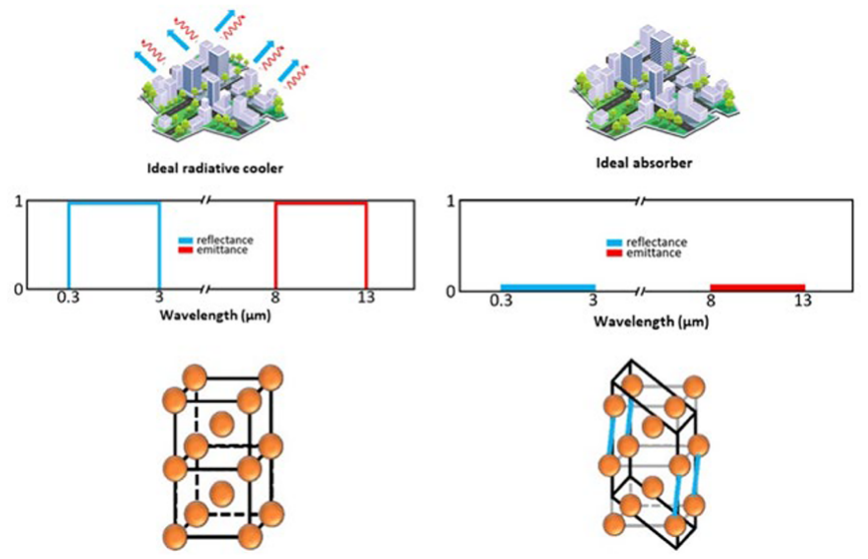

Traditional cooling and heating systems in residential buildings account for more than 15% of global electricity consumption and 10% of global emissions of greenhouse gases. Daytime radiative cooling (DRC) is an emerging passive cooling technology that has garnered significant interest in recent years due to its high cooling capability. It is expected to play a pivotal role in improving indoor and outdoor urban environments by mitigating surface and air temperatures while decreasing relevant energy demand. Yet, DRC is in its infancy, and thus several challenges need to be addressed to establish its efficient wide-scale application into the built environment. In this Perspective, we critically discuss the strategies and progress in materials development to achieve DRC and highlight the challenges and future paths to pave the way for real-life applications. Advances in nanofabrication in combination with the establishment of uniform experimental protocols, both in the laboratory/field and through simulations, are expected to drive economic increases in DRC.

The rapid expansion of urban areas has intensified the urban heat island (UHI) phenomenon,^1^ characterized by higher temperatures in urban environments compared to surrounding rural areas. This overheating is influenced by the thermal and radiative properties of urban surfaces.^2^ Consequently, there has been a significant scientific effort to develop passive strategies to improve the local microclimate by leveraging the intrinsic properties of specific materials.^3^

Since the development of the first daytime radiative cooler (DRC) in 2014, which demonstrated subambient surface temperatures under direct sunlight, daytime radiative cooling has emerged as a promising passive cooling approach for the built environment and UHI mitigation.^4^ In the past decade, numerous research articles have reported on the successful development of DRCs for built environment applications.^5^ However, several challenges, including high costs and the lack of scalable solutions, continue to hinder their exploitation in real-life applications.

Existing dielectric-based materials have been explored for their optical properties, geometries, and architectures, yielding interesting cooling efficiencies.^6^ However, many solutions offer static responses without seasonal modulation of cooling performance and are generally limited to white reflecting surfaces. Materials that exhibit environment-responsive optical and electronic behavior can more effectively address urban cooling needs at various temperatures and enhance aesthetic acceptance.^7,8^

In addition to in-lab and in-field investigations, simulations that incorporate modern modeling techniques can play a critical role in scaling up DRC technology. Finite-Difference-Time-Domain (FDTD) and Rigorous-Coupled-Wave Analysis (RCWA) are currently popular numerical methods for optimizing DRCs’ optical properties at the material scale.^9^ Limited studies have also examined the performance of DRCs at the building scale in terms of cooling capacity and energy savings, while only a few research groups are investigating DRCs at the larger neighborhood and city scales.^10^

Successful deployment of DRC technology in real-world applications necessitates a comprehensive understanding of the current state of the art. However, the lack of standardized experimental protocols in both laboratory and field investigations, resulting in potential biases in the evaluation of cooling demonstrations, makes it difficult to identify key innovations and research gaps. Therefore, establishing standardized experimental protocols for laboratory and field investigations is essential to evaluate the performance of materials, improve their reliability and comparability, and facilitate the evaluation of DRC technology in real contexts.

In this Perspective, we delve into the evolving DRC strategies, placing particular emphasis on their material properties and potential applicability in the built environment. Our objective is to shed light on how this progress can enhance passive cooling solutions, thereby augmenting both indoor and outdoor comfort while aiding UHI mitigation. Indeed, we recognize the crucial link between DRC technologies and UHI mitigation, and our analysis is centered on the exploration and optimization of material-scale advances in DRC. These strides in materials science could prove instrumental in real-world UHI mitigation applications.

By pushing DRC technologies toward optimization for maximum efficiency, scalability, and adaptability, we believe that they can contribute substantially to reducing urban heat accumulation, a central facet of UHI mitigation. Furthermore, enhancing the understanding and control of the optical and electronic properties of materials involved in DRC will enable harnessing these passive cooling techniques more effectively. We also discuss the exciting future prospects for material developments in DRC applications, extending our perspective to include recommendations for advancing research and refining testing protocols to support real-life applications. Thus, we also discuss the exciting future prospects for material developments in DRC applications, extending our perspective to include recommendations to advance research and refine test protocols to support real-life applications.

## 1 Daytime Radiative Cooling for the Built Environment

Radiative cooling (RC) occurs naturally at night when a surface emits thermal energy within the atmospheric window (AW, 8–13 μm), where the atmosphere is nearly transparent. However, the AW transparency is highly sensitive to local water vapor contents. In fact, in regions characterized by high humidity, the water vapor content tends to be elevated, which can significantly lower the transmittance in the AW. This reduced transmittance acts as a substantial limiting factor because the higher the local water vapor content, the less effective the RC effect becomes due to the reduction of long-wave radiation released into space. Yet, it is important to acknowledge that the benchmark evaluation of DRC strategies typically considers a standard transparency of the AW. This standardization allows for the controlled comparative analysis necessary for material optimization and design improvements of various DRC materials and geometries under consistent boundary conditions.

Daytime radiative cooling (DRC) is facilitated by specially designed materials that (i) exhibit high reflectivity within the short-wave radiation spectrum (SR, 0.01–2.5 μm) and (ii) exhibit high emissivity within the infrared spectrum (IR, 2.5–100 μm), particularly within the AW spectrum. Ideally, a DRC material should have 100% reflectivity within the SR spectrum and an emissivity efficiency of 1 in the AW region of the spectrum only (and 0 in the other spectral regions). Most DRCs reported in the literature have reflectivities greater than 80%.

Approaches to controlling emissivity are divided between selective DRCs (SDRCs) and broadband DRCs (BDRCs). SDRCs aim to achieve the maximum possible subambient surface temperature reduction by minimizing parasitic losses due to heat conduction and convection, necessitating proper insulation. In contrast, BDRCs, which emit across the entire IR spectrum, do not require insulation and transfer their cooling effects through convection. Both SDRCs and BDRCs have demonstrated subambient temperatures under direct sunlight, making them viable solutions for a range of applications, including passive cooling of buildings, UHI mitigation, solar cell optimization, the clothing industry, electricity generation, and water harvesting.

Actually, many tested cooling materials present an intrinsic wide emittance spectrum in the IR range, facilitating their use as BDRC materials in large-scale contexts, such as in building infrastructures. On the other hand, the selective emittance spectrum of SDRCs, while also effective, requires a more tailored application approach. Therefore, although the optimization of both SDRCs and BDRCs is important in the advancement of DRC technologies, the innate broadband emittance of BDRCs lends them a certain advantage when considering large-scale, real-world applications. Yet, the selective traits of SDRCs can also be instrumental under specific conditions and can achieve unprecedented cooling effects. Optimizing both of these types of DRCs ensures a comprehensive and adaptive approach to advancing passive cooling techniques, creating a more robust and versatile arsenal for UHI mitigation strategies.

In this context, DRCs are considered the next generation of built environment materials with the potential to regulate and improve urban environments, mitigating UHI effects currently experienced in cities worldwide, and improving indoor and outdoor thermal comfort.

## 2 Strategies Proposed for Daytime Radiative Cooling

**Figure 1:**
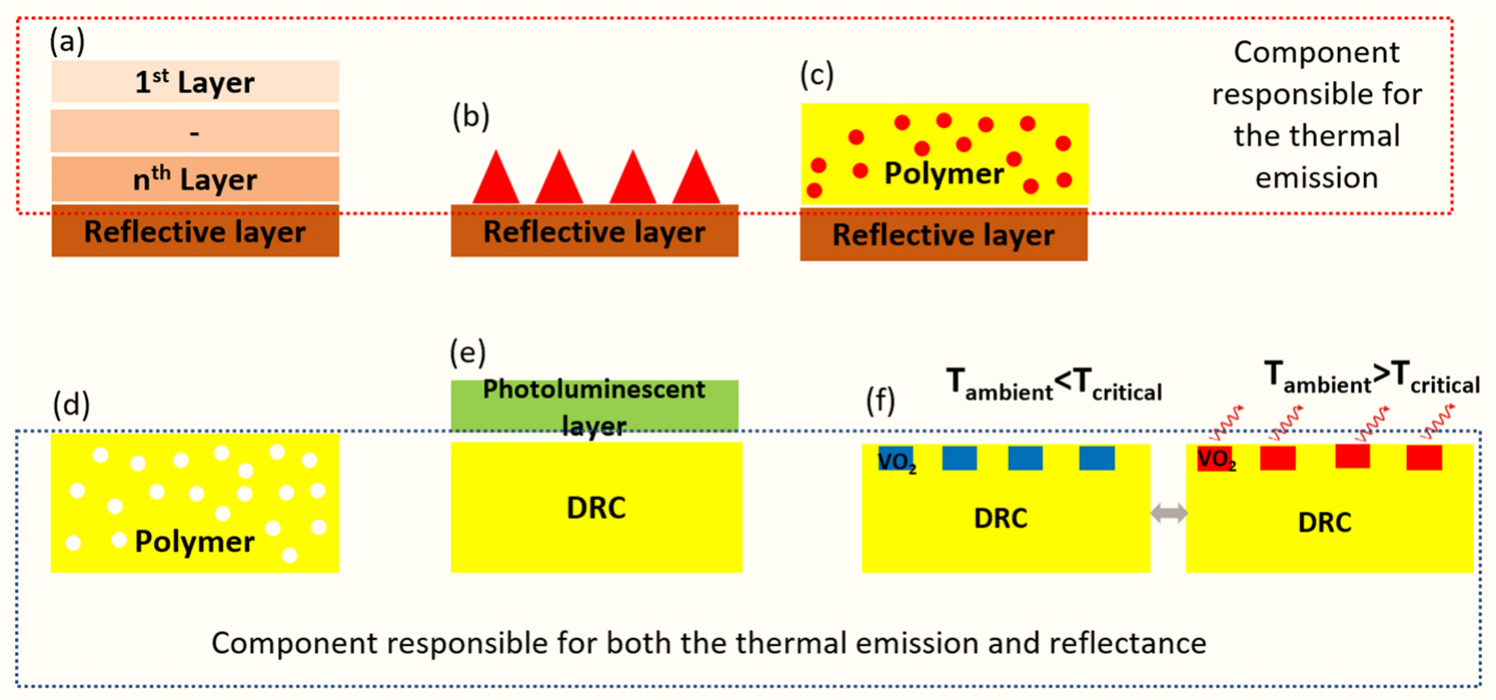
DRC types: (a) multilayer DRCs, (b) metamaterial DRCs, (c) DRC structures with randomly distributed particles, (d) porous DRCs, (e) colored DRCs, and (f) adaptive DRCs.

Apart from being characterized by a selective or broadband emittance, DRCs reported in the literature can be classified into six main categories (Figure 1): (i) multilayer DRCs, (ii) metamaterial DRCs, (iii) DRC structures with randomly distributed particles, (iv) porous DRCs, (v) colored DRCs, and (vi) adaptive DRCs. (The main component of colored and adaptive DRCs can be any type of the first three DRC categories.)

Multilayer and metamaterial DRCs represent the first generation of developed DRCs. Their design is primarily a combination of two crucial parts: the upper part is short-wave-transparent and is highly absorptive and emissive for long-wave radiation, and the lower part effectively reflects short-wave radiation. Multilayer DRCs utilize the principles of interference, diffraction, and refraction to control the optical properties of their layers. The choice of materials and their precise structuring in layers, in terms of number and thickness of the layers, determine the ability of these systems to selectively reflect and release thermal radiation almost exclusively within the AW. In the case of metamaterial DRCs, their controlled space arrangement in ordered patterns confers selective properties to govern the propagation of electromagnetic waves. This fine-tuned control is harnessed to direct thermal radiation effectively within the AW. In fact, metamaterial DRCs represent a significant step forward in the development of RC technologies, primarily due to their sophisticated nanostructured upper layer. These material architectures, produced through advanced nanofabrication techniques, are meticulously designed and prepared to emit primarily within the AW, enhancing the cooling efficacy.

DRC structures with randomly distributed particles (such as titanium dioxide, zinc oxide, silicon nitride, barium sulfate, and calcium carbonate nanoparticles) bear a certain resemblance to multilayer and metamaterial DRCs. These structures also consist of two essential components: an emissive layer and a layer responsible for short-wave reflection. The emissive component of these structures is generally a polymer-based material whose emittance is regulated by the size of the particles and their concentration. The particles embedded within the polymer matrix enhance the IR emissivity of the material. By leveraging the properties of these particles and carefully tuning their size and concentration within the polymer matrix, it is possible to adjust the spectral characteristics of the DRC to achieve an optimal cooling performance.

Porous DRCs represent another category of DRCs, wherein the incorporation of air voids within a polymer matrix results in distinctive optical characteristics. In terms of reflectance, the presence of air voids significantly influences the backscattering of incoming short-wave solar radiation. The disparity in the refractive index between the air voids and the surrounding polymer leads to multiple scattering events, effectively reducing the absorption of solar radiation and enhancing the reflectance. The air voids also enhance the IR emissivity of the porous DRCs. The polymer–air interface within these materials can promote the emission of thermal radiation in the IR range, especially within the AW.

The magnitude of the short-wave radiation reflectivity impacts the color of a surface. The lighter the color is, i.e., the more it approximates white, the higher the short-wave reflectance is. As a result, developing colored surfaces, which are important for real-life built environment applications, without compromising their reflectivity has always been an important challenge for the scientific community. The same challenge is also true for DRCs, taking into consideration their already complex nature. Recently, a small number of research groups reported on the development of colored DRCs that, apart from a bottom reflective layer and an intermediate emissive layer, comprise additionally an upper photoluminescent layer, as described in section 2.2.

Wide-band-gap inorganic semiconductor materials have been investigated for cooling purposes; these materials have a negligible absorption cross section in the visible (VIS) region and relevant mid-IR extinction coefficients, resulting in highly selective emissivity. Metal oxides were used to prepare the DRC layered structures (Table 1). The use of several layers for passive RC was developed by Raman et al.,^4^ who proposed a device composed of seven layers of alternating HfO_2_ and SiO_2_ on top of a 200 nm Ag layer and a silicon wafer used as substrate; they measured a temperature reduction of 4.9 °C on a winter day in California. Jeong et al. tested a deposition made up of eight TiO_2_ and SiO_2_ alternating multilayers,^11^ highlighting the high costs of multilayered nanoscale depositions through controlled methodology, like electron beam. Other metal oxides have been used in layered or nanostructured strategies,^12−15^ evidencing the possibility to achieve a temperature reduction ranging from 2.5 to 22.3 °C.

**Table 1:** Summary of Materials with Passive Radiative Cooling Effects

**Material**	**Structure/Geometry**	**Temperature Reduction**	**Ref**
SiO_2_/HfO_2_	Multilayers	4.9 °C	(4)
TiO_2_/SiO_2_	Multilayers	7.2 °C	(11)
Porous anodic aluminum	Porous membrane on Al	2.6 °C	(12)
HDPE/Cr_2_O_3_/TiO_2_	Composite artificial lawn	12.7 °C	(13)
Al_2_O_3_	Microfibers	22.3 °C	(14)
3D porous cellulose acetate/SiO_2_	Composite membrane	6.2 °C	(15)
SiO_2_/Si_3_N_4_	Multinanolayers	11 °C	(16)
4A Zeolite	PVDF used as binder	15 °C (compared to Al)	(17)
		4 °C (compared to TiO_2_)	
AlPO_4_	PVDF used as binder (87 wt% AlPO_4_)	4.2 °C	(18)

In addition to metal oxides, other dielectric materials were tested to obtain DRC. Yao et al.^16^ reported the use of SiO_2_/Si_3_N_4_ in near-perfect selective photonic crystal layers. 4A zeolite was also presented as a good candidate for cooling.^17^ AlPO_4_ was reported by Li et al.^18^ to reach a temperature drop of 4.2 °C lower than the ambient temperature and of 4.8 °C compared to commercial heat insulation coating.

However, depending on the microclimate boundary conditions, the application of materials that reject radiation and heat from the built environment results in a winter penalty. Their high reflectance and emittance during the cold period of the year lead in many cases to a rebound effect that may aggravate indoor comfort and exacerbate energy consumption related to heating of indoor spaces. Thus, a DRC strategy needs to be optimized taking into tight consideration the environment boundary conditions.

Looking to conventional cool materials (CMs), thermochromic properties are probably the most attractive solution for counteracting the winter penalty. Thermochromic materials adapt their optical and electronic behaviors with respect to a threshold ambient temperature value (transition temperature). For ambient temperatures above the transition temperature, their surface is highly reflective, while below the threshold value, they absorb VIS frequencies and their surface color becomes darker.

**Figure 2:**
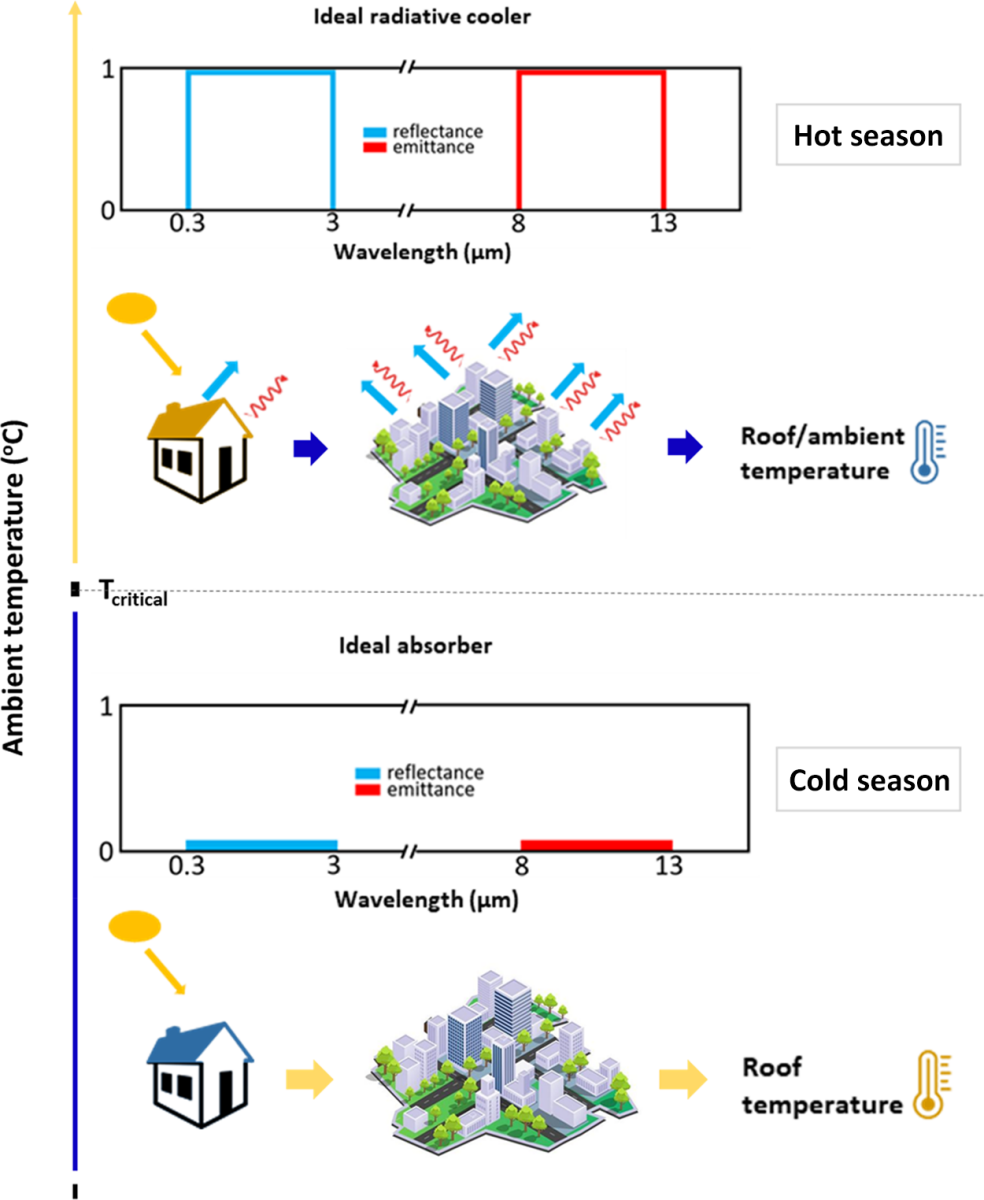
Yearlong configuration of an ideal DRC for all-season thermal regulation.

This temperature-controlled optical behavior ensures adaptive function of the surface realized with thermochromic materials and ensures an efficient implementation of DRCs throughout both hot and cold periods of the year (Figure 2). Yet, making DRCs adaptive to ambient temperature is a rather sophisticated problem due to their complex nature and the need to modulate accordingly both reflectance and emittance. As a result, only a small number of studies have been reported, to date, on adaptive DRCs.

### 2.1 Adjustable DRCs with the Thermochromic Principle

While the broader field of DRC includes promising alternatives such as photo- and electrochemically induced switchable radiative coolers, the focus of this Perspective is specifically on DRC technologies that operate without the need for additional energy input and are temperature dependent. These passive, thermally triggered solutions are of particular interest in the context of sustainable urban heat island (UHI) mitigation and contribute significantly toward the decarbonization of the built environment. These technologies align with climate goals for reducing the energy consumption and associated carbon emissions in urban landscapes. Therefore, these strategies can contribute significantly to the creation of more sustainable, efficient, and resilient urban environments.

Numerous examples of temperature-adaptive coolers are found in the literature, such as liquid crystals,^19,20^ leuco dyes,^21,22^ or phase-changing materials. However, organic materials, despite their adaptive properties, are generally less suitable for outdoor applications due to their lower resilience to mechanical and meteorological stress. Therefore, inorganic materials are often preferred for passive RC applications.

Among the inorganic materials considered for these purposes are titanium dioxide, zirconium oxide, and zinc sulfide. These materials are white at room temperature but can turn yellow under extremely high temperatures.^23−25^ While these materials offer certain benefits, their color-changing temperatures are impractical for most real-world applications. Similarly, mercury(II) iodide displays a reversible phase transition at 126 °C, transitioning from red to yellow. However, again, this transition temperature is far outside of practical ranges.

Within this context, vanadium dioxide (VO_2_) has emerged as a promising alternative. This material undergoes a phase transition at a more reasonable temperature of about 68 °C, changing from a monoclinic to a rutile phase.^26,27^ This transition is accompanied by a change in the material’s electronic and optical properties: VO_2_ acts as a semiconductor below the transition temperature, with a bandgap of 0.66 eV, and as a metal in the rutile phase. These unique properties make VO_2_ an attractive candidate for integration into building materials, providing heat retention during colder months and a highly reflective surface during warmer seasons, effectively contributing to both heating and cooling strategies.

**Figure 3:**
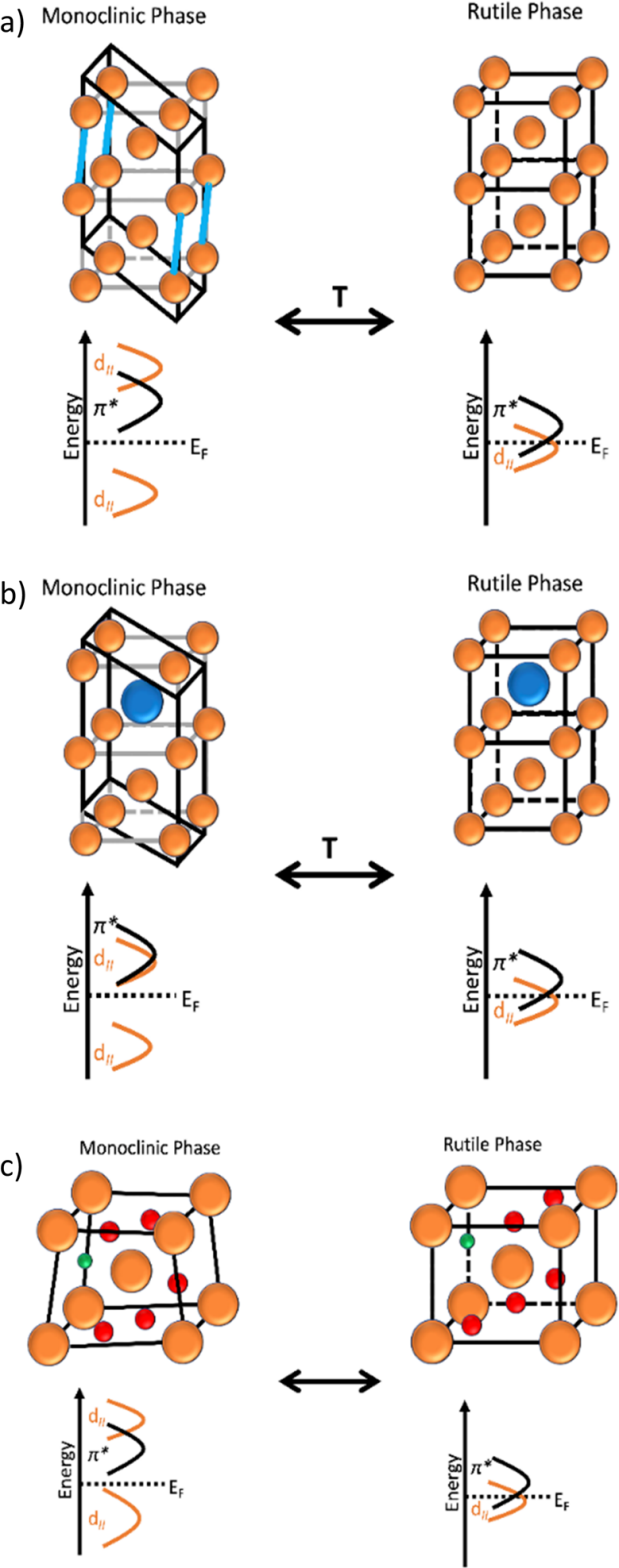
Schematic representation of transition from monoclinic to rutile phase in VO_2_ (a), in cation-doped VO_2_ (b), and in anion-doped VO_2_ (c) together with their electronic band structures. Orange spheres represent V^4+^ ions, larger blue spheres are cation dopants, red spheres are oxygen atoms, and green spheres are anion dopants. In (a) bonds are highlighted to better display the distortion between monoclinic and rutile phases; in the representations reported in (a) and (b), oxygen atoms are omitted for the sake of clarity.

The rutile phase is based on a simple tetragonal lattice (space group *P*4_2_/*mnm*) where the V atom is surrounded by an edge-sharing octahedron of oxygen atoms. The monoclinic phase (space group *P*2_1_/*C*) derives from a distortion and doubling in size of the rutile phase, and in the structure, two different V(IV)–V(IV) distances can be found (0.265 and 0.312 nm, respectively),^28^ as shown in Figure 3a.

Despite those advantages, some drawbacks must be overcome to make this material commercially attractive: the harsh conditions to prepare VO_2_ and the still too high transition temperature.^29−33^ Concerning the first point, the syntheses reported in the literature require first strong oxidant, i.e., hydrogen peroxide, followed by reducing agents, i.e., hydrazine; then, high temperatures to form the powder are needed, usually ranging from 240 to 280 °C. The combination of these factors makes this material unsuitable for large-scale synthesis and application. Regarding the second point, the semiconductor-to-metal transition of VO_2_ occurs at too high a temperature to make the material suitable for real-life applications. One of the possible routes to lower this temperature is to induce a destabilization of the monoclinic phase and a decrease in the transition temperature;^34,35^ it is widely accepted that the traces of V(III) and/or V(V) in the structure cause a disruption of the V(IV)–V(IV) units, decreasing the transition temperature.

However, the main strategy to systematically cope with the problem is the use of dopants inside the VO_2_ matrix. Tungsten is reported to efficiently decrease the transition temperature of VO_2_, and literature reports document a drop in transition temperature even to 24.2 °C.^35−38^ W(VI) enters in the VO_2_ lattice and substitutes V(IV) species; this substitution results in a modification of the length of metal–oxygen–metal chains. W(VI) is larger than V(IV) ions, so the length of V–O–W chains is shorter than that of V–O–V chains, and they induce a destabilization of the monoclinic phase,^36^ as depicted in Figure 3b. In addition, Tan et al.^39^ demonstrated that, in W-doped VO_2_, tungsten preserves a symmetric tetragonal-like structure, acting as a nucleus for the transition of the nearby asymmetric monoclinic VO_2_ lattice toward rutile phase.

Taking advantage of surface patterning procedures, Tang and co-authors^40^ fabricated a temperature-adaptive radiative coating (TARC) based on W-doped VO_2_ on a dielectric surface able to set the transition temperature at 22 °C. In this work, the authors demonstrated that the TARC could be used in all seasons for building thermal regulation as it achieves a self-switching of thermal emittance from 0.20 to 0.90 in the AW.

Nevertheless, tungsten is a rare heavy metal, and since 2020 it has been included in the list of critical raw materials by the EU. For this reason, new dopants that could perform as well as tungsten are required. Other elements were tested as cationic dopants that substitute vanadium in VO_2_, such as magnesium,^41^ antimony,^42^ terbium,^43^ molybdenum, and niobium.^44^ When magnesium enters into the lattice, it causes deformation of the V–V chain due to the creation of oxygen vacancies. Magnesium acts also as a network modifier, lowering the network coordination.^45^ Concerning antimony, terbium, and molybdenum, the drop in the transition temperature was attributed to the same phenomenon occurring in W-doped VO_2_. When niobium is used as dopant, it substitutes the V(IV) ion, causing a charge transfer forming V(III) and Nb(V).^46^

Another viable alternative is the doping of VO_2_ with anion species that replace oxygen, such as fluorine^39^ and nitrogen.^47^ Fluorine is reported to inject electrons into the V 3d valence band, causing a reduction from V(IV) to V(III) through charge transfer, whereas nitrogen creates or increases hole carrier concentration, triggering an earlier transition in VO_2_ (Figure 3c). Table 2 summarizes the data collected on doped VO_2_.

**Table 2:** Summary of Doped VO_2_ Materials and Their Passive Radiative Cooling Properties

**Material**	**Dopant (%)**	**Transition Temperature (°C)**	**Maximum Temperature Reduction (Δ*T*, °C)**	**Ref**
W-VO_2_	1–2.5	24.2	43.8	(35)
W-VO_2_	6–50	46.7	21.3	(36)
W-VO_2_	1–4	53	14	(37)
W-VO_2_	1–2.5	0.1	54.9	(38)
Mg-VO_2_	2.5–7.2	45	23	(41)
W-F-VO_2_	0–3 (both W and F)	0	68	(48)
Sb-VO_2_	0.48–33.10	62	7	(42)
Tb-VO_2_	1–10	60	7.5	(43)
Mo-VO_2_	3–11	32	36	(44)
Ni-VO_2_	4–11	34	34	(44)

VO_2_ would become a benchmark material for passive RC applications if its metal-to-insulation transition temperature could be lowered to the 25–40 °C range using sustainable and abundant elements such as iron, copper, silicon, and zinc. Moreover, the combination of two or more dopants could be tested to determine their effects on the VO_2_ transition. Lastly, the synthetic methodologies to prepare VO_2_-based materials should be improved to reduce the reaction temperatures and the use of reactants.

### 2.2 Photoluminescent Materials for Passive Cooling

Conventional apparatuses use white or reflecting (silver-like) materials, not taking into consideration the aesthetic of the final product. To become a viable alternative, passive radiative coolers need to be colorful, even though this means the absorption of a fraction of UV or VIS radiation and a possible radiative heat loading.^49^ A mitigating solution to the heat loading is the use of photoluminescent materials; in particular, materials able to absorb UV and/or VIS light and relax through a radiative deactivation path are excellent compromises to dissipate the absorbed radiation energy.^50,51^ Indeed, photoluminescence always occurs at energies lower than those of the absorbed light. This means that the emitted radiation has a lower energy content compared with the absorbed fraction, entailing a cooling of the surface. Son et al.^52^ demonstrated the cooling effect achieved through the use of a photoluminescent layer, and they proposed that the wavelength conversion from which luminescence originates (UV radiation to VIS luminescence) avoids the reflective layer of their apparatus absorbing UV radiation, causing a decrease in temperature. However, this field is in its early stages, and few examples are reported in the literature.

In 2020, Garshasbi et al.^7^ demonstrated that quantum dots can be used as additives to the state-of-the-art coatings to enhance passive RC; the measured data indicate up to 2 °C temperature reduction when CuInS_2_/ZnS core/shell quantum dots (maximum absorption and emission at 544 and 639 nm, respectively, resulting in 340 meV Stokes shift) with quantum yield of around 50% are used to coat the exposed surface. Furthermore, the developed thermal balance model for fluorescent materials highlights the importance of maximizing the photoluminescence quantum yield and the number of photons absorbed (within the solar range).

**Figure 4:**
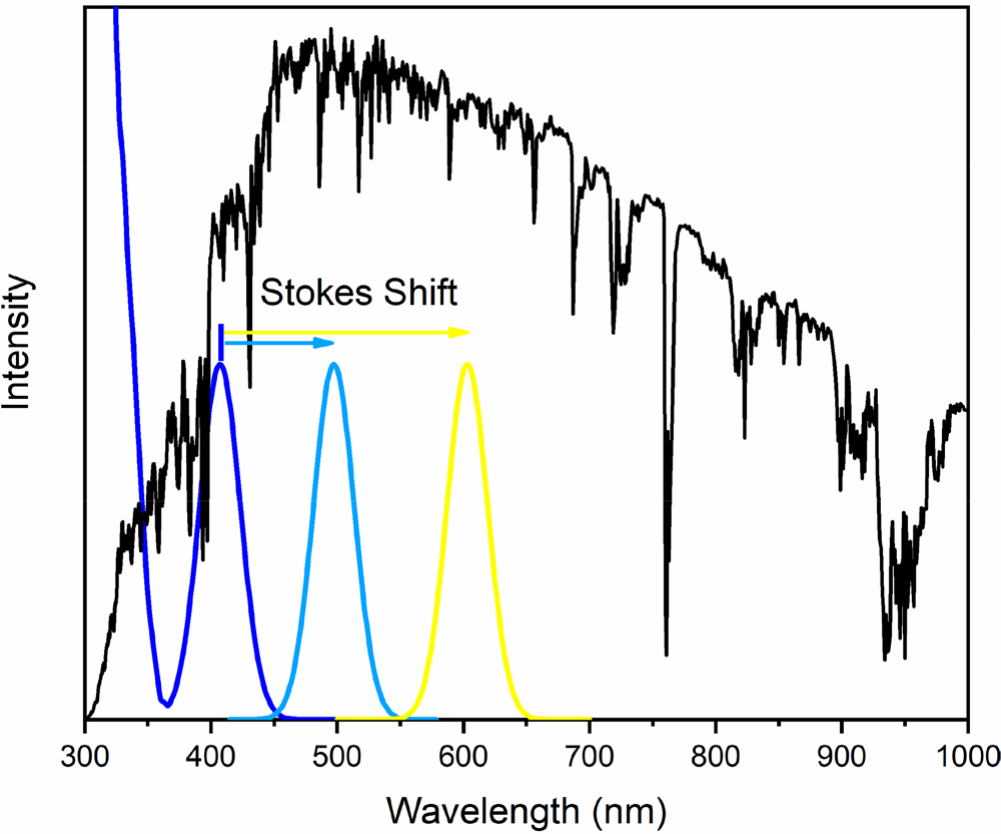
Schematic representation of photoluminescence upon radiation absorption (blue) with narrow (cyan) and wide (yellow) Stokes shifts. The solar emission spectrum (black) is reported for clarity.

Wang et al.^53^ reported the use of perovskite quantum dots (CsPbX_3_, X = Br, I), which can be tuned to have green, yellow, and red colors and have a drop in temperature between 5.4 and 2.2 °C. Always taking advantage of perovskite properties, Son et al.^54^ prepared a core–shell system where they encapsulated CsPbBr_3_ and CsPbBr_*x*_I_3–*x*_ perovskite nanocrystals in SiO_2_ to enhance the stability of perovskites in humid conditions. The authors tested nanocrystals having the emission tuned in green and red colors (540 and 650 nm, respectively). The different luminescence properties of the core–shell nanocrystals are paired with different radiation harvesting in the VIS region; in particular, one sample absorbs until 520 nm, while the sample with red emission presents an absorption spectrum that spans the entire VIS region. Interestingly, the surfaces with nanocrystals showed an efficient daytime subambient cooling of 3.6 and 1.7 °C for the green- and red-emitting materials, respectively; the assignment of the cooling to photoluminescence-assisted RC is validated by the comparison of surface temperatures with those observed with commercial non-luminescent paints with similar colors/absorption features. Thus, the generation of luminescence upon radiation absorption instead of heat enhances the cooling performances. Furthermore, it is interesting to highlight that the data and the developed thermal balance model indicate that the role of photoluminescent materials in RC applications could be further enhanced by employing materials with higher luminescence yields and larger Stokes shifts, as depicted in Figure 4.

Indeed, by using luminophores with wide Stokes shifts, the fraction of solar radiation absorbed can be limited, enhancing the cooling effects due to the smaller amount of energy of the radiation re-emitted by them in the environment, as shown by the green-emitting perovskites.^54^

Among photoluminescent materials, one particularly appealing type can result in the exploitation of compounds with long-lasting or persistent luminescence, the so-called persistent phosphors. The persistency of the afterglow luminescence, which can last for hours,^55^ is beneficial for sustainable lightning or signs;^56^ this possibility makes these materials exploitable not only for building but also for built pavements. The inclusion of phosphors in the top layer of a bilayer RC coating was demonstrated to be a good strategy to obtain a colored passive radiative cooler;^57^ the authors tested four different phosphors which displayed temperature reductions between 0.6 and 2.7 °C. In a recent paper, it was demonstrated that phosphorescent components possess good surface temperature reduction during hot days, maintaining their luminescent properties.^8^ Generally, inorganic persistent phosphors consist of inorganic matrices containing emitting centers; in many reports, the emitting centers are rare earth cations,^55,58^ which have narrow and efficient luminescence but limited absorption cross sections and relevant sustainability concerns. In recent years, persistent phosphors with engineered structural defects able to trap charge carriers have been investigated.^59,60^

At present, phosphors are widely investigated and tested because they fulfill the requirement of large Stokes values to help the thermal balance analysis, but they fail to efficiently harvest solar radiation to ensure high brightness, especially in the range where the human eye presents the highest sensitivity. Very recently, this issue has been faced by preparing mixtures of fluorescent and phosphorescent materials. Materials with luminescence passed on by spin-allowed transitions (fluorescence) are able to ensure efficient and fast transitions (absorption and emission), while compounds having spin-forbidden or trapped charge carrier transitions provide a long-lasting emission. The optimized combinations of fluorescent and phosphorescent materials have proven to take advantage of energy-transfer processes to guarantee reasonable luminance and longer afterglow,^61^ along with the possibility to spectrally tune the emitted radiation.

Nevertheless, it should also be emphasized that, in designing photoluminescent materials for passive cooling, their capacity to generate phonon modes in the IR must be controlled. The occurrence of absorptions in the 0.3–2.5 μm and/or the 8–13 μm regions can limit the energy radiated by the cooling devices in AW. Jeon etal.^62^ reported the use of CsPbBr_3_ perovskite nanocrystals able to absorb UV–blue radiation without generating IR phonons. This observation highlights the urgency to characterize the phononic modes of luminescent materials with promising properties for cooling applications, like perovskites.^63^

## 3 Scaling-Up DRCs for Real-Life Applications

Up to now, we have only considered the optical and thermal performance of the DRCs, but other features such as the manufacturing costs, the scalability, and the long-lasting performance of high quality under real-life boundary conditions as well as their environmental footprint must be taken into consideration.

In fact, a wide real-life application requires an efficient DRC application covering large areas, such as roofs and facades. Nevertheless, the vast majority of relevant studies report on the development and test of small-scale specimens covering an area of a few centimeters. Therefore, the scaling-up of these DRC specimens should take into account the main prerequisites for real-life applications: high cooling capacity in the long run in conjunction with low-cost manufacturing and high compatibility. For instance, the complex design architecture and multistep microfabrication process of multilayer and metamaterial DRCs that typically comprise electron beam evaporation, magnetron sputtering, plasma-enhanced chemical vapor deposition, photolithography, and reactive ion etching render these DRCs a rather non-cost-effective solution that cannot be scaled up for large-scale applications. On the other hand, polymer technology is, at the present, more mature; hence, the manufacturing processes of polymer-based DRCs comprise relatively simpler techniques such as roll-to-roll fabrication, infiltration, electrospinning, centrifugation, and simple mixing and coating, rendering these DRCs more cost-effective and their scalability more feasible.

Under this scenario, DRC structures with randomly distributed particles and porous DRCs should be considered better candidates for built environment applications due to their polymer-based nature. Utilizing low-cost nanoparticles is pivotal for reducing the costs of DRC structures with randomly distributed particles, while choosing the appropriate size can further enhance their performance for real-life applications. For instance, unlike single-size particles, hierarchical-sized particles scatter over a broad spectrum and hence can decrease the corresponding manufacturing cost due to less need for precise particle size control.

The development of DRCs that do not comprise a reflective layer, but instead simultaneously perform high short-wave reflectance and IR emittance due to air voids, can, on one hand, further reduce the fabrication costs.^64^ In fact, polymer-based systems with air voids present a cost-effective and easily manufacturable solution, making them particularly appealing for large-scale applications, where cost considerations play a significant role. Nevertheless, their inherent trade-offs, especially concerning durability, cannot be disregarded.^65^ The potential impacts of mechanical stresses and weathering on these systems over time can influence their performance and longevity.^66^ This aspect becomes crucial when deliberating their suitability for real-world applications, necessitating careful evaluation of their long-term efficiency against initial cost advantages.

In addition, a large-scale and real-life implementation of DRCs requires, in many cases, colored DRCs. In fact, due to either aesthetic preferences or aesthetic prerequisites, an efficient production of DRCs having desired colors will pave the way for their wide real-life application. Inevitably, though, the addition of an upper photoluminescent layer will add extra costs that need to be taken into account. Nevertheless, the current research dedicated to the optimization of photoluminescent materials is rapidly evolving and is expected to bring about not only a cost-effective solution but also highly efficient photoluminescence emittance that may result in further enhancement of the subambient cooling.

## 4 Devising a Research Framework for DRCs

Manufacturing efficient DRCs for real-life applications is, indeed, a multifaceted challenge. Yet, apart from explicitly tailoring the core optical properties of the DRCs, further feedback-loop investigation protocols comprising in-lab, in-field, and simulation experimental procedures should be uniformly adopted by the scientific community to ensure the appropriate scaling-up of DRCs (Figure 4).

In fact, it is necessary to establish experimental protocols and standards for each scale of relevant analyses, i.e., material, building, neighborhood, city, and even global scale. Under this scenario, experiments both in-lab and in-field should go hand in hand with detailed numerical simulations and should analytically assess the specific implementation boundaries by investigating the effects on (i) surface temperature reduction, (ii) energy savings of the building envelope, and (iii) ambient air temperature reduction. Further proof-of-concept evaluation should be carried out with pilot-case projects under realistic boundary conditions representing real-scale conditions.

Concerning material-scale investigations, the number of layers, the size and distance of the particles/voids, and the corresponding nanometric architecture in conjunction with the morphology of the finishing and surface should be thoroughly devised. Concerning building-scale investigations, the orientation and type of the building, its skin characteristics, the type of its use, possible aesthetic prerequisites, neighboring buildings and architecture, and the local climate zone microclimate boundaries should be taken into account. Concerning neighborhood, city, or bigger scales of investigation, the corresponding (local) climate zone boundaries, the urban architecture, proximity to sea/desert, sky view factor, and atmospheric boundaries should be taken into account.

At a material scale, there are several simulation tools that can be used for applying techniques such as finite-difference-time-domain (FDTD) or rigorous-coupled-wave analysis (RCWA) in order to optimize the structure and optical characteristics of DRCs. Monte Carlo simulations can also be applied for evaluating photons’ propagation within DRC structures with randomly distributed particles and porous DRCs. These outcomes can be subsequently coupled with both building simulation tools and detailed urban canopy models for evaluating DRCs’ performance on larger urban scales.

The development of established 3-D simulation protocols is a crucial step forward in the evaluation of DRCs. These protocols should be informed by relevant experimental procedures conducted within sophisticated climate chambers as well as carefully curated outdoor areas or pilot case studies. These environments allow for the testing of DRC technologies under varied and dynamic conditions that more closely resemble their intended real-world applications.

However, one significant challenge that persists is the accurate simulation of atmospheric transparency within the AW during these in-lab tests. Replicating the intricate effects of the AW within the controlled confines of a climate chamber is a complex task. Therefore, future research efforts should be dedicated to developing robust in-lab experimental protocols that can more effectively simulate and incorporate the influence of the AW. Once this challenge can be overcome, any DRC type could be tested for almost any microclimate boundaries to maximize its cooling capacity, opening new doors for efficient scaling-up.

Another significant challenge in advancing DRC technologies and evaluating their performance is the lack of standardized in-field experimental protocols. The absence of such consistent testing methods results in a high degree of variability in the experimental conditions across studies, rendering it challenging to compare their outcomes.

The thickness of samples, their inclination, the insulation applied, their altitude with respect to sea level, the positioning of sensors, the choice of reference material, and the optical and thermal characteristics of nearby materials are just a few of the numerous factors that tend to vary from one study to another. These discrepancies can profoundly affect the reported performance of DRCs, adding an extra layer of complexity to their evaluation.

For instance, the thickness of the DRC samples can directly influence their thermal and radiative properties, thereby affecting their cooling performance. Similarly, the inclination of the samples could affect their exposure to solar radiation, thus impacting the energy balance and the resulting cooling effect. The insulation applied to the DRC samples can also alter their thermal interaction with the surrounding environment, which in turn can impact the measured cooling effect. Furthermore, altitude, which determines the atmospheric pressure, can also influence radiative transfer and thus cooling performance.

Meanwhile, the positioning of the sensors and the choice of reference materials in the experiments can affect the accuracy and reliability of the measurements. Additionally, the optical and thermal characteristics of nearby materials can influence the local microclimate, adding another level of variability to the experimental results.

Given these complexities, there is a pressing need to establish and adopt consistent in-field experimental protocols for evaluating DRCs. This standardization would allow for more meaningful comparisons between studies, providing a solid foundation for the advancement of DRC technologies. Moreover, understanding these variables and their effects on DRC performance will also help guide the design and optimization of DRC materials and strategies, ultimately contributing to more effective and scalable UHI mitigation solutions.

**Figure 5:**
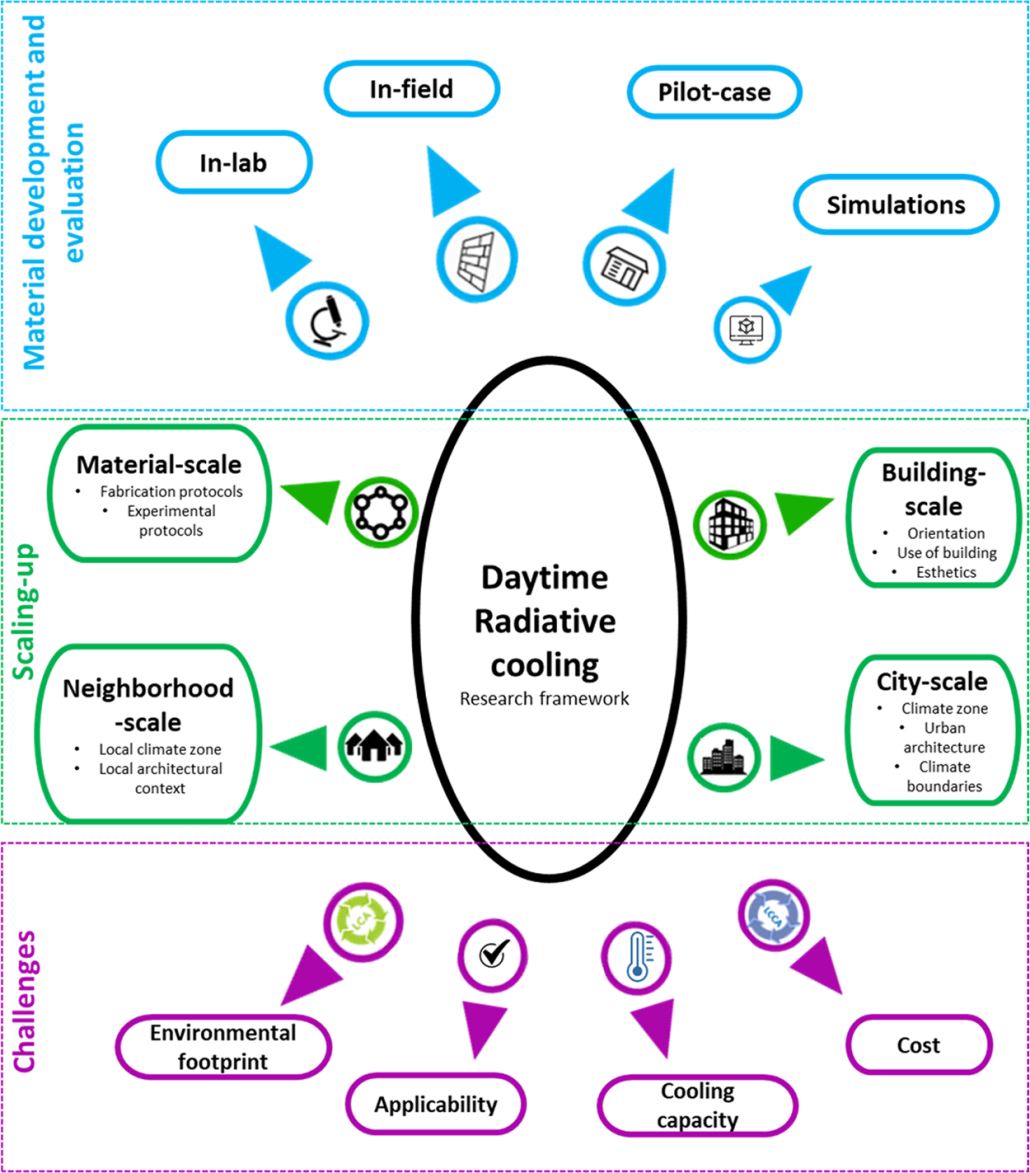
Research framework for scaling-up DRCs.

Their applicability in a variety of urban contexts and architectures would be further endorsed through the exploitation of mature technologies such as polymer-based technologies that can simultaneously decrease manufacturing costs. Finally, the environmental footprint of DRCs should be assessed at each step of their lifecycle to ensure their alignment with sustainability goals related to the built environment. Figure 5 summarizes our research framework for scaling-up DRCs.

## 5 Outlook

In summary, recent advancements in daytime radiative coolers (DRCs) for built environment applications have shown promise as passive cooling strategies aimed at enhancing indoor and outdoor comfort while mitigating the urban heat island (UHI) effect. This paper offers readers an updated perspective and deeper comprehension of DRCs. However, the current state-of-the-art has yet to reach the benchmarks required for practical, large-scale applications and corresponding commercialization.

For the successful implementation of DRCs in real-world settings, several critical factors must be addressed. First, the effectiveness of selective or broadband DRCs in the built environment must be thoroughly examined in terms of surface temperature reduction and UHI mitigation potential. Second, prioritizing and leveraging advanced technologies, such as polymer-based approaches, is essential for developing cost-effective solutions. Third, large-scale experimental assessments, both in-field and numerical, are required to determine the viability of DRCs in urban environments, taking into account various implementation scales. Additionally, the establishment of standardized experimental protocols coupled with simulation frameworks is crucial for providing continuous feedback and optimizing DRCs in relation to the specific constraints of the implementation area. This includes considering both existing and new building envelopes. Moreover, the environmental impact of DRCs, which is currently lacking in the literature, must be meticulously evaluated throughout their production and analysis stages.

In this Perspective, we also highlight the great advantage of VO_2_ that is its transition temperature, which is the closest to room temperature among the other inorganic thermochromic materials. A combination of VO_2_ and photoluminescent materials could be a competitive and feasible alternative to materials used for passive radiative cooling (RC), as they would benefit from the features of both. Nevertheless, some considerations must be done. In fact, for real applications, the properties of the materials must remain the same as those studied in laboratory. Thus, interfaces and how the photoluminescent material is applied/deposited on surfaces become crucial points to avoid the disruptive effects of optically mismatched interfaces. Non-optimized surfaces or interfaces between the different components of the device could lead to scattering, reflection, and dissipation effects with unoptimized geometries that will decrease their efficiency.^67−69^ The main drawbacks in passive RC applications could be reduced photoluminescence for photoluminescent materials and an impossibility to emit the radiation in the atmospheric window for VO_2_.

A wide array of emerging technologies and disciplines, ranging from material science to urban architecture, must be synergistically integrated to scale up DRCs for effective real-life applications capable of enhancing urban environments and mitigating UHI. Consequently, it falls upon the scientific community, industry stakeholders, and policymakers to tackle the ongoing challenges associated with DRCs and pave the way for their cost-effective implementation in diverse urban contexts and architectural designs worldwide.
